# High admission rates and heavy inpatient service costs of urban tuberculosis patients in eastern China

**DOI:** 10.1186/s12913-019-3892-9

**Published:** 2019-01-18

**Authors:** Yang Zhou, Cheng Chen, Hui Jiang, Hong-Qiu Pan, Li-Mei Zhu, Wei Lu

**Affiliations:** 1 Department of Chronic Disease Control and Prevention, Centers for Disease Control and Prevention of Jiangsu Province, 172 Jiangsu Road, Nanjing, 210009 Jiangsu Province People’s Republic of China; 2 Centers for Disease Control and Prevention of Zhenjiang City, Zhenjiang, Jiangsu Province People’s Republic of China; 3 No.3 Hospital of Zhenjiang City, Zhenjiang, Jiangsu Province People’s Republic of China

**Keywords:** Tuberculosis, Inpatient, Financial burden, China

## Abstract

**Background:**

Tuberculosis patients often experience hospitalization. Inpatient services may result in high medical expenditures. It is important to explore the hospitalization rates of tuberculosis patients and the potential factors that are associated with admission rates and inpatient service expenditures.

**Methods:**

Data from patients diagnosed and treated at the No.3 hospital of Zhenjiang City from Apr. 2014 to Mar. 2015 were obtained. Univariate and multivariate statistical analyses were applied for the analysis of potential factors associated with admission rates, average length of stay and cost.

**Results:**

A total of 356 tuberculosis patients were treated at the No.3 hospital of Zhenjiang City. A total of 221 of the 356 patients were hospitalized. Sputum smear test results and type of health insurance were the potential factors associated with hospitalization rates. The average admission was (1.26 ± 0.64) per patient. The average length of stay of inpatients was 29.99 ± 25.83 days. Age, occupation, and sputum smear test were related to the average length of stay. The average total cost to inpatients was 13007.91 ± 5205.58 CNY. The sputum smear test results, type of health insurance, occupation and age were the main potential factors associated with TB inpatient expenditures.

**Conclusions:**

The admission rate of tuberculosis patients was high. Despite advances in TB insurance policies, there were substantial costs associated with TB diagnosis and treatment. TB patients still face a heavy financial burden. Health care providers should revise the service package and reform the health insurance regulations to ensure that TB patients receive appropriate care.

**Electronic supplementary material:**

The online version of this article (10.1186/s12913-019-3892-9) contains supplementary material, which is available to authorized users.

## Background

China is one of the “high burden countries” of tuberculosis (TB), there were 889,000 new TB cases in 2017 according to the estimates of the World Health Organization (WHO). China has the second highest TB epidemic rate in the world [1].

TB remains a serious public health issue and social problem in many developing nations [2]. The Chinese government attaches much importance to TB control. Since the 1990s, the Chinese government has implemented the National Tuberculosis Control Program (NTP), the DOTS strategy, free TB diagnosis and treatment, and DOTS-plus to constantly enhance TB control measures [3].

TB has been regarded as a “poverty-related disease” due to its association with poverty and malnutrition [4]. Poverty can decrease the rate of treatment success [5], and TB will also aggravate poverty. In China, suspected tuberculosis patients are provided free diagnosis and anti-tuberculosis treatment, including free chest X-ray examinations, sputum smear tests and designated first-line anti-tuberculosis drugs. However, some patients still cannot afford the share of medical and non-medical costs [6].

Recently, tuberculosis management models have been changing in China. The diagnosis and treatment of TB patients have been transferred from the Centers for Disease Control and Prevention (CDC) to “designated hospitals”. Each county has one designated hospital for TB treatment. The designated hospital is usually a public hospital in the county, and most of them are the largest healthcare provider in the county [7]. In China, patients are more likely to seek medical service in the public hospital, including suspected and confirmed TB patients. The 2000 national TB survey reported that 91% of TB patients visited public hospitals first to treat TB related symptoms [8]. Additionally, TB patients receive better diagnosis and treatment in the designated hospitals, and can also increase adherence to anti-tuberculosis treatment. However, the designated hospital model also has its own disadvantages; patients may not receive the free diagnosis and anti-tuberculosis treatment or they may experience over-examination and a high rate of hospitalization due to poor hospital staff training for TB technical specifications and the economic incentive of the designated hospital. Research from Dr. Li [9] revealed that the rate of hospitalization increased significantly from 62.3 to 98.0%, and the ALOS increased 2 to 4 days after the diagnosis and treatment of TB patients transferred to the designated hospitals. These resulted in a greater financial burden on patients and reduced the likelihood of treatment adherence. Some patients default because of the high financial burden of TB [10].

In 2013, China launched a collaboration with the Bill and Melinda Gates Foundation TB Programme. Under this programme, three project sites, including Zhenjiang City Jiangsu Province, implemented a new policy to standardize TB diagnosis and treatment at the designated hospital and improve reimbursement rates for TB patients. In this paper, we evaluated the effectiveness of the programme. We analysed the admission rates and economic burden of TB patients in Zhenjiang City, and we also explored the potential factors that are associated with high admission rates and inpatient service costs.

## Methods

### Study design

In 2004, we began to explore the designated hospital management model in Jiangsu Province, and until 2012, all province counties implemented the designated hospital model. Since 2009, the Bill and Melinda Gates Foundation, in collaboration with the China National Health and Family Planning Commission/China CDC, has been implementing an innovative programme for TB control and prevention in four Chinese cities [11]. In 2013, the second phase of this programme was initiated. Zhenjiang City is one of the three project cities. The programme aims to establish comprehensive TB control models that can standardize clinical behaviour, decrease the rate of hospitalization and increase the reimbursement rate on TB patients.

The targets of the programme are as follows: i) maintain the rate of hospitalization under 30% and ii) implement case-based payments and develop a standard service package for inpatients. The service package is developed according to examination procedures, including sputum tests, sputum cultures, drug susceptibility tests, chest X-rays, physical examinations, biochemical examinations, and electrocardiogram examinations, and treatment for TB patients, including adverse reaction treatments, ward services, etc. The total cost of the service package was capped at 8000 CNY (Chinese Yuan). For services in the package, patient payment capped at a maximum of 20% of charges or 1600 CNY, and health insurance covered 80% of charges. Patients whose health insurance covered over 80% of the package followed the original payment policy. The assessment of the project was conducted in 2015. In this study, we analysed the admission rate and inpatient cost of No. 3 hospital of Zhenjiang City (the designated hospital of Zhenjiang City).

Jiangsu Province lies in eastern China, adjacent to Shanghai City. There are 13 cities in Jiangsu Province. In 2015, the population was 79.76 million and the gross domestic product (GDP) was 70116 billion CNY, GDP per capita was 88085 CNY (14143USD). Zhenjiang City lies in the southeast of Jiangsu Province. In 2015, the population was 3.16 million and the GDP was 3502 billion CNY. GDP per capita was 110440 CNY (17732 USD), ranked fifth in Jiangsu Province.

### Data collection

#### Medical records from TB designated hospitals

Active TB patients who lived in Jingkou District, Runzhou District, Dantu District, or Xin District that were diagnosed and treated in the No.3 hospital of Zhenjiang City from Apr. 2014 to Mar. 2015 were all participants, including new patients and retreatment patients. MDR-TB, rifampicin-resistant TB, extrapulmonary TB, and tuberculous pleurisy patients were excluded. Data were obtained from the China TB Management Information System (TMIS)*,* inpatient records were obtained from the Hospital Information System (HIS)*,* expenditures of inpatient cases obtained from Hospital Reimbursement System (HRS). All subjects gave their informed consent for inclusion before they participated in the study.

#### Expenditure associated with TB treatment

Fees for services covered by the free-TB service policy were directly deducted and not recorded in the patients’ medical records. The total costs were medical inpatient expenses related to TB diagnosis and treatment. The out-of-pocket (OOP) costs were the total costs calculated by excluding reimbursement through medical insurance. Inpatients who were hospitalized for diseases other than tuberculosis were not counted, the medical expenses incurred by the subjects not related to tuberculosis during hospitalization were also not included in the expenses. The costs are expressed in the Chinese Yuan (CNY). In 2015, 1 CNY was equal to 0.16 USD.

#### Admission indicators

All of the subjects in this study were TB patients, and the primary diagnosis for hospital admission was TB. Hospital admission was also assessed according to the following indicators: i) Patients with severe complications; ii) Patients with serious adverse reactions requiring further hospitalization; iii) Patients requiring surgery; and iv) Patients with other conditions requiring admission to the hospital as determined by physicians.

### Statistical analysis

Univariate and multivariate analyses were applied to analyse the data. Patient’ names were used as a key variable to link the data from TMIS, HIS and the Hospital Reimbursement System. Chi-square tests were used to identify factors associated with admission rates. ANOVA was used to find differences in hospitalization times and the average length of stay (ALOS). Costs were presented as the means (standard deviation, SD) and medians (percentile 25 and percentile 75, p25 and p75) to allow for the expected positively skewed distributions. The Mann-Whitney U and Kruskal-Wallis H tests were used to compare the cost differences. A binary logistic regression was used to explore the association between the characteristics of TB patients and the occurrence of hospitalization, and a generalized linear regression was used to explore the association between the characteristics and the medical cost for TB inpatient care. Statistical significance was set at 5%. The statistical package SPSS 20.0(Texas, USA) was used to analyse the data.

## Results

### Demographic and social characteristics

Three hundred fifty-six TB patients (Additional file 1) were diagnosed and treated at the No.3 hospital of Zhenjiang City from Apr. 2014 to Mar. 2015. Table 1 shows that approximately 75.8% of patients were men, and 24.2% of patients were women. A large majority were aged 45 years and older (66.8%). Among the 356 patients, 43.2% were manual labourers. A large majority were new cases, and only 14.6% were previously treated cases. More than half of the TB patients were native to Zhenjiang City, and 46.9% of the patients were smear-positive cases.

**Table 1:** Demographic and social characteristics of the 356 TB patients (N/%)

Variables	*n*	%
Gender		
Male	270	75.8
Female	86	24.2
Age		
< 30	70	19.7
30~44	48	13.5
45~59	99	27.8
60~	139	39.0
Occupation		
Mental labourer^a^	34	9.6
Manual labourer^b^	154	43.2
Retire^c^	146	41.0
Unknown	22	6.2
Patient category		
New cases	304	85.4
Previously treated cases	52	14.6
Residence		
Native	203	57.0
Migration in China	153	43.0
Sputum smear test		0.0
Negative cases	189	53.1
Positive cases	167	46.9

^a^Mental labourer, including students, teachers, health workers and governmental personnel

^b^Manual labourer, including farmers, rural migrant workers, shepherd

^c^Retire including retire and housekeeping

### Admission rate and potential factors associated with the admission rate

#### Admission rate

Patient’ names were used as key variables to link the data from TMIS and HIS. We found that 221 patients among the 356 patients were hospitalized (Additional file 2). According to Table 2, most of the inpatients were male (*P* = 0.017). There were significant differences in the hospitalization rates between smear-positive and smear-negative patients (*P* < 0.001). The hospitalization rate of patients covered by student health insurance was significantly higher than that of patients covered by the Urban Resident Basic Medical Insurance scheme (URBMI) and the Urban Employee Basic Medical Insurance scheme (UEBMI) or without insurance.

**Table 2:** Analysis on hospitalization rates of 356 TB patients

Variables	Total patients	Inpatients	Hospitalization rate (%)	Chi-Square	*P*-value
Gender					
Male	270	177	65.6	5.740	0.017
Female	86	44	51.2		
Age					
< 30	70	40	57.1	4.444	0.217
30~44	48	26	54.2		
45~59	99	69	69.7		
60~	139	86	61.9		
Occupation					
Mental labourer	34	22	64.7	2.691	0.442
Manual labourer	154	89	57.8		
Retire	146	94	64.4		
Unknown	22	16	72.7		
Patient category					
New cases	304	183	60.2	3.129	0.077
Previously treated cases	52	38	73.1		
Residence					
Native	203	130	64.0	0.771	0.380
Migration in China	153	91	59.5		
Sputum smear test					
Negative cases	189	92	48.7	30.738	0.000
Positive cases	167	129	77.2		
Health Insurance					
URBMI	136	80	58.8	7.893	0.048
UEBMI	161	96	59.6		
Student	13	12	92.3		
Without insurance	46	33	71.7		

#### The potential factors associated with admission rates

A binary logistic regression was used to explore the association between six characteristics of TB patients (gender, age, occupation, patient category, residence, sputum smear test and health insurance) and the occurrence of hospitalization by computing odds ratios (ORs). Table 3 shows that sputum-smear positive patients were more likely to have higher hospitalization rates than sputum smear-negative patients (OR = 3.576, *P* = 0.000). Significant differences were found in the hospitalization rates in health insurance. TB patients covered by student insurance and patients without insurance were more likely to be hospitalized.

**Table 3:** Multivariate analysis of the potential factors influence hospitalization rates of the 356 TB patients

Independent variables	β	SE	*P*-value	OR	95% *CI*
Gender (Compare with female)	0.412	0.277	0.137	1.510	0.877–2.599
Age (Compare with <30)			0.167	1.000	
Age 30–44	0.038	0.440	0.931	1.039	0.438–2.460
Age 45–59	0.757	0.394	0.054	2.132	0.986–4.611
Age 60+	0.380	0.383	0.322	1.462	0.689–3.099
Occupation (Compare with Mental labourer)			0.304	1.000	
Manual labourer	0.352	0.533	0.509	1.422	0.501–4.037
Retire	0.707	0.542	0.192	2.027	0.701–5.864
Unknown	1.018	0.718	0.156	2.768	0.678–11.297
Patient category (Compare with new patients)	−0.043	0.380	0.909	0.958	0.455–2.015
Residence (Compare with native)	−0.038	0.249	0.878	0.962	0.591–1.567
Sputum smear test (Compare with Negative)	1.274	0.261	0.000	3.576	2.143–5.968
Health insurance (Compare with UEBMI)			0.012	1.000	
URBMI	0.147	0.280	0.598	1.159	0.670–2.005
Student	3.397	1.177	0.004	29.865	2.976–1299.701
Without insurance	0.834	0.415	0.045	2.302	1.021–5.192

In Table 4, 221 inpatients experienced 279 hospitalizations with an average of 1.26 ± 0.64 admissions per patient. A total of 19.9% of inpatients were hospitalized at least once. The ALOS of inpatients was 29.99 ± 25.83 days, and 34.4% of inpatients were hospitalized over 4 weeks. There was a significant difference in the hospitalization times associated with the type of health insurance (*P* = 0.005). A univariate analysis identified several features related to ALOS, including age, occupation, and sputum smear status. The ALOS of positive cases was longer than that of negative cases.

**Table 4:** Factors related to the average times of hospitalization and ALOS of the 221 inpatients

Variables	Inpatients	Times of hospitalization	F^a^	*P*-value	ALOS	F^a^	*P*-value
Gender							
Male	177	1.25 ± 0.60	0.148	0.701	30.72 ± 27.08	0.880	0.349
Female	44	1.30 ± 0.76			27.12 ± 20.17		
Age							
< 30	40	1.18 ± 0.45	0.459	0.711	33.68 ± 23.17	4.464	0.004
30~44	26	1.23 ± 0.43			35.72 ± 24.24		
45~59	69	1.26 ± 0.50			34.59 ± 34.87		
60~	86	1.31 ± 0.83			23.28 ± 16.19		
Occupation							
Mental labourer	22	1.45 ± 1.14	1.097	0.351	29.81 ± 26.59	3.639	0.013
Manual labourer	89	1.19 ± 0.45			27.68 ± 16.58		
Retire	94	1.29 ± 0.65			29.09 ± 28.44		
Unknown	16	1.25 ± 0.45			47.90 ± 40.06		
Patient category							
New cases	183	1.24 ± 0.64	1.279	0.259	28.98 ± 23.64	1.860	0.174
Previously treated cases	38	1.37 ± 0.63			34.38 ± 33.68		
Residence							
Native	130	1.32 ± 0.74	2.901	0.090	29.69 ± 22.71	0.058	0.810
Migration in China	91	1.18 ± 0.44			30.46 ± 30.28		
Sputum smear test							
Negative cases	92	1.22 ± 0.59	0.792	0.374	22.76 ± 11.55	15.409	0.000
Positive cases	129	1.29 ± 0.67			34.83 ± 31.13		
Health Insurance							
URBMI	80	1.14 ± 0.38	4.465	0.005	30.95 ± 32.05	1.529	0.207
UEBMI	96	1.42 ± 0.83			27.74 ± 21.08		
Student	12	1.42 ± 0.67			26.88 ± 18.32		
Without insurance	33	1.06 ± 0.24			37.74 ± 26.89		

^a^ANOVA test

### Inpatient economic burden and the potential factors associated with medical expenditure

#### Inpatients’ economic burden

We obtained medical expenditure records for 141 of the 221inpatients (Additional file 3). As shown in Table 5, the average (median, IQR) total costs of service package was 7219.78(7503.03, 6251.10–7942.20) CNY, and the OOP costs were 1320.24 (1433.13, 1179.55–1570.76) CNY. The median (IQR) ratio of OOP costs to total costs was 20.00% (19.15–20.00). Except for the costs of the service package, patients still had to pay a substantial proportion medical expenditures occurred out of the service package, which was almost half of the total costs. The median (IQR) ratio of OOP costs to total costs out of the service package was 41.88% (26.23–55.57). The average (median, IQR) total cost to inpatients was 13007.91(11674.20, 10054.08–15579.08) CNY.

**Table 5:** Medical expenditure of 141 inpatients

Costs	mean	SD	median	25%	75%
Service package					
Total	7219.78	1900.81	7503.03	6251.10	7942.20
OOP	1320.24	332.88	1433.13	1179.55	1570.76
OOP/Total	18.58	3.30	20.00	19.15	20.00
Out of service					
Total	5788.13	4814.67	4127.52	2865.05	7633.46
OOP	2198.35	1760.18	1738.12	870.68	2836.55
OOP/Total	40.02	16.64	41.88	26.23	55.57
Total costs					
Total	13,007.91	5205.58	11,674.20	10,054.08	15,579.08
OOP	3518.60	1868.56	3125.85	2203.58	4222.57
OOP/Total	26.89	8.65	27.10	20.41	33.22

#### The potential factors associated with the high expenditure of inpatients

In Table 6, the expenditures of native patients were higher than the costs of migration in China (*P* = 0.035) in the service package. Out of the service package, a univariate analysis showed several features related to OOP expenditures, including occupation and type of health insurance. There was a different reimbursement rate outlined by the reimbursement policy. The expenditure for manual labourers, which was always farmers, was higher than in other occupations, and the reimbursement rate was usually lower than in other populations. The total OOP situation was the same.

**Table 6:** Factors related to medical expenditure of 141 inpatients

Variables	*n*	Costs in service package(IQR^a^)	OOP in service package(IQR)	Costs out of service package(IQR)	OOP out of service package(IQR)	Total costs(IQR)	Total OOP(IQR)
Gender							
Male	112	7550.06 (6381.08~7942.03)	1445.46 (1179.35 ~ 1571.85)	4139.26 (2888.82~7496.82)	1735.98 (870.03~2837.59)	11,699.02 (10,261.18~15,704.9)	3158.78 (2244.72~ 4227.44)
Female	29	7318.50 (6031.39~ 7970.65)	1324.29 (1142.21~ 1573.14)	3819.53 (2809.05~ 8399.07)	1909.02 (968.99~ 2821.23)	11,357.23 (9167.00~ 15,211.91)	3059.63 (1881.25~ 4242.81)
Z^b^		−.0924	−.0780	−0.061	−0.189	−0.648	−0.423
P		0.356	0.435	0.951	0.850	0.517	0.672
Age							
< 30	19	7731.34 (6900.82~ 8000.00)	1546.27 (1380.16~ 1600.00)	3873.73 (2501.13~ 6478.41)	1656.26 (781.53~ 2739.06)	11,292.02 (9634.83~ 15,955.36)	3060.25 (2327.80~4326.82)
30~44	16	7388.26 (5686.05~ 7924.16)	1455.68 (1137.21~ 1584.83)	3413.56 (3132.89~ 5082.48)	1535.78 (1141.18~ 2433.21)	11,075.71 (10,303.37~13,308.02)	2907.03 (2324.57~3948.44)
45~59	47	7582.86 (6621.44~7941.30)	1443.92 (1292.09~1566.97)	5404.18 (3819.53~9366.75)	2325.03 (1440.76~3581.04)	12,384.00 (10,691.04~16,136.52)	3604.12 (2815.03~ 4997.82)
60~	59	7318.50 (6008.03~7931.95)	1325.39 (1130.19~1565.42)	3745.21 (2849.05~7097.73)	1558.50 (600.83~2766.46)	11,572.33 (9070.85~15,250.24)	2763.16 (1944.84~3915.32)
Chi-square ^c^		1.371	5.110	5.883	5.944	4.355	6.476
P		0.712	0.164	0.117	0.114	0.226	0.091
Occupation							
Mental labourer	13	7503.03 (5524.31~8000.00)	1500.61 (1104.86~1600.00)	3788.99 (1702.54~4225.39)	1300.91 (734.8~1620.99)	10,800.82 (7465.5~14,169.68)	2776.70 (1943.31~3064.02)
Manual labourer	64	7478.35 (6346.45~7951.91)	1453.94 (1259.45~1587.55)	5025.66 (3366.65~7898.30)	2436.18 (1600.63~3974.04)	11,809.49 (10,588.68~15,798.30)	3727.75 (2829.70~5393.51)
Retire	55	7505.63 (6000.00~7931.95)	1379.26 (1040.92~1566.97)	3718.17 (2775.14~8864.82)	1397.58 (571.14~2124.04)	11,824.17 (10,158.03~16,077.79)	2713.60 (1797.64~3548.55)
Unknown	9	7295.57 (6301.51~7856.34)	1433.94 (1179.28~1547.69)	3968.55 (2386.22~4568.42)	1731.26 (749.84~2651.82)	10,700.03 (9356.06~ 12,012.02)	3272.37 (1702.72~4095.52)
Chi-square ^c^		0.092	3.565	5.308	19.050	4.472	18.395
P		0.993	0.312	0.151	0.000	0.215	0.000
Patient category							
New cases	123	7449.71 (6227.50~7938.81)	1429.23 (1170.93~1563.12)	4167.60 (2994.47~7701.50)	1909.02 (871.97~2834.46)	11,679.87 (10,536.20~15,698.47)	3153.33 (2271.07~4212.82)
Previously treated cases	18	7715.12 (6328.78~8000.00)	1513.47 (1255.86~1600.00)	3685.84 (1740.12~7498.85)	1572.54 (574.44~3063.01)	11,168.72 (7343.38~15,399.63)	2885.47 (1928.92~4660.40)
Z^b^		−0.615	−0.923	−1.199	−0.883	−1.118	−0.735
P		0.539	0.356	0.231	0.377	0.263	0.462
Residence							
Native	85	7568.60 (6670.75~8000.00)	1477.88 (1241.26~1599.14)	4167.60 (2863.47~7784.43)	1829.43 (894.02~2919.84)	11,824.17 (10,674.78~15,892.04)	3202.07 (2293.47~4218.16)
Migration in China	56	7225.72 (5890.99~7859.26)	1379.71 (1102.42~1561.69)	3967.10 (2888.82~7261.65)	1735.98 (810.35~2817.46)	11,540.42 (8880.55~15,132.54)	3017.66 (1975.05~4261.99)
Z^b^		−2.112	−2.134	−0.282	−0.177	−1.298	− 0.725
P		0.035	0.033	0.778	0.860	0.194	0.469
Sputum smear test							
Negative cases	68	7225.72 (6002.01~7856.24)	1401.84 (1174.55~1553.33)	3846.63 (2605.13~5532.08)	1671.18 (719.36~2498.17)	11,260.25 (9007.33~12,974.59)	3017.66 (1918.94~3714.91)
Positive cases	73	7600.00 (7000.00~8000.00)	1459.11 (1192.32~1589.37)	5030.39 (3205.26~9360.52)	2009.50 (1060.22~3303.87)	12,384.00 (10,790.90~17,247.91)	3316.15 (2286.02~4769.94)
Z^b^		−1.892	−0.898	−2.921	−1.696	−3.569	−1.646
P		0.058	0.369	0.003	0.090	0.000	0.100
Health Insurance							
URBMI	61	7169.72 (5861.54~7968.72)	1405.92 (1150.56~1578.59)	4447.89 (2962.81~7496.26)	2387.33 (1552.39~3966.36)	11,586.84 (9270.80~14,764.00)	3736.14 (2543.41~5261.62)
UEBMI	70	7642.62 (6930.02~7941.75)	1452.36 (1199.08~1569.67)	4194.55 (2977.00~8368.94)	1513.24 (700.39~2482.56)	11,942.67 (10,697.78~16,081.56)	2910.21 (2078.23~3718.84)
Student	10	7201.93 (4652.65~7814.37)	1440.39 (930.53~1562.88)	3140.65 (1390.16~3937.07)	1263.56 (555.61~1676.73)	10,085.52 (6641.69~12,457.86)	2670.89 (1534.47~3042.38)
Chi-square ^c^		3.627	1.024	4.850	13.552	6.344	10.933
P		0.163	0.599	0.088	0.001	0.042	0.004

^a^*IQR* Interquartile range

^b^Mann-Whitney U test

^c^Kruskal-Wallis H test

We also tried to find associations between the characteristics of TB patients and medical expenditures. Generalized linear regression analysis was used, in which the log of the medical expenditures was the dependent variable as the data were skewed. Table 7 indicates that total costs were positively correlated with occupation and sputum smear-positive patients. The expenditure of smear-positive patients (12384.00 CNY) was higher than that of smear-negative patients (11260.25 CNY). The results also revealed that type of health insurance, occupation and age were potentially associated with total OOP costs. The expenditure for manual labourers was much higher than that of other occupations. Another factor that could have influenced the OOP expenditure of inpatient services was type of health insurance. Patients covered by URBMI (3736.14 CNY) paid more than patients covered by UEBMI (2910.21 CNY) and student insurance (2670.59 CNY), because out of the service package, the reimbursement rate of URBMI was lower than that of UEBMI and student insurance.

**Table 7:** Generalized linear regression analysis of potential factors influence medical costs

Variables	Total costs			Total OOP costs		
	β	SE	*P*-value	β	SE	*P*-value
Intercept	8.944	0.286	0.000	7.359	0.411	0.000
UEBMI	0.398	0.238	0.094	0.771	0.341	0.024
URBMI	0.350	0.241	0.146	0.924	0.347	0.008
Student	0^a^			0^a^		
Male	0.003	0.072	0.972	−0.041	0.103	0.691
Female	0^a^			0^a^		
Mental labourer	0.281	0.229	0.220	0.473	0.330	0.151
Manual labourer	0.247	0.125	0.048	0.390	0.180	0.030
Retire	0.219	0.125	0.079	0.069	0.180	0.699
Unknown	0^a^			0^a^		
New cases	0.164	0.091	0.069	0.138	0.130	0.288
Previously treated cases	0^a^			0^a^		
Native	0.038	0.061	0.531	0.064	0.088	0.468
Migration in China	0^a^			0^a^		
Negative cases	−0.200	0.061	0.001	−0.064	0.088	0.465
Positive cases	0^a^			0^a^		
Age	− 0.003	0.002	0.107	−0.008	0.003	0.005
Scale	0.111^b^	0.013		0.229^b^	0.027	

Dependent variable:total costs/total OOP costs

Model (intercept): health insurance, gender, occupation, patient category, residence, sputum smear test and age

a Set to zero because this parameter is redundant

b Maximum likelihood estimate

## Discussion

This study explored the admission rate of TB patients and analysed the expenditures of inpatients in Zhenjiang City. The results revealed that the admission rate of TB patients was high. TB inpatients had to bear a heavy economic burden, even with the free TB policy in China. With this policy, sputum smear tests, free chest X-ray examinations and first-line anti-tuberculosis drugs are provided to TB patients [12–14]. The study also revealed that the sputum smear test results, type of health insurance, occupation and age were the main potential factors associated with TB patient admission rate and inpatient expenditures.

Outpatient treatment is recommended by the WHO for non-complicated tuberculosis patients [15]. In this study, the admission rate of TB patients was 62.1%, which exceeded the target of the programme and higher than in the previous study [16]. In this article, a binary logistic regression analysis showed that sputum smear-positive patients and students were more likely to have higher hospitalization rates. First, sputum smear-positive patients often were seriously ill or accompanied by complications, and patients were more likely to be hospitalized. Second, with the decrease in government funding for public hospitals in China, insurance and patient payments are the main sources of income for health facilities [17]. Physicians employed by hospitals are rewarded with bonuses and promotions based on the profits they generate by admitting patients, recommending diagnostic tests, and prescribing medications. This undoubtedly encourages physicians to increase the hospitalization of TB patients. Third, considering that tuberculosis is an infectious disease, parents and school administrators worried about the spread of TB infection within the family and in the school. Therefore, they prefer the students to be admitted to the hospital. Patients without insurance also had a high hospitalization rate. The target hospitalization rate of less than 30% was restricted to patients with insurance. Patients without insurance were not controlled by the health department.

In this study, we found that the ALOS of positive TB patients was 34.83 ± 31.13 days, which was longer than the ALOS of negative TB patients (22.76 ± 11.55 days). Previous studies have shown that smear-positive TB patients admitted to hospitals after 14 days of anti-tuberculosis treatment had a sputum negative conversion rate of 61.15%; there still had 38.85% of patients had sputum smear-positive results [18]. Considering infection control, doctors tend to extend the length of hospital stay for smear-positive patients [19, 20].

Physicians should restrict hospitalization and shorten the length of stay according to the admission indicators. Outpatient treatment not only deceases the economic burden but also avoids nosocomial infections and the spread of MDR-TB. However, unnecessary hospitalization has been reported and leads to higher inpatient expenses [21]. Hospitalization may be responsible for the high financial burden on TB patients [12, 22, 23]. The medical expenditure of inpatients was 3.28 times that of outpatients [24]. In 2007, a study showed that ALOS was 28.8 days, and the average cost of inpatients was 7725.6 CNY [25]. In our study, the ALOS was 29.99 days, 34.4% of inpatients were hospitalized over 4 weeks. Long hospital stays and unnecessary examinations have caused a heavy burden of TB [26].

There are three basic health insurance schemes in China. UEBMI, a compulsory scheme, was established for the urban residents who formally work. URBMI, a voluntary scheme, was established for the rest of urban residents without formal jobs or who are unemployed, such as children, elderly, and the unemployed young. The New Rural Cooperative Medical Scheme (NCMS), a voluntary scheme, was established for rural areas. URBMI and NCMS have a lower reimbursement rate for tuberculosis. The Zhenjiang City government has combined the NCMS and URBMI into URBMI. Under the free-TB service policy for tuberculosis, basic health insurance plays a small role in TB treatment [27]. Basic health insurance usually does not cover much of the outpatient service or provides a low reimbursement rate [28], which may drive TB patient hospitalization rates. The basic health insurance policy increased the OOP costs of TB and even resulted in CHE [29]. A study funded by GFATM revealed that only 41% of the counties covered the outpatient costs by basic health insurance, and the reimbursement rate was approximately 50%. The amount of reimbursement was approximately 400 CNY [30]. Another study in eastern China showed that the reimbursement rate covered by NCMS was only 25.1% [31]. To some extent, the lower reimbursement rate leads to the higher admission rate. Some TB patients will accept hospitalization because of reimbursement [9]. Currently, in most places of China, basic health insurance partly reduced the economic burden on TB, though patients still cannot bear OOP expenditures [32].

During the implementation of the Gates Foundation programme, Zhenjiang City increased the reimbursement rate for tuberculosis outpatients and inpatients to 80%. However, this reimbursement rate was limited to the service package. Constraints of the service package, the inpatients in Zhenjiang City benefited approximately 70% reimbursement rate indeed. We observed that the average total cost in the service package was 7219.78 CNY, and the ratio of OOP costs to total costs was 18.58%, which achieved the goals of the programme. However, hospitals often provided TB patients with extra services and medicines beyond the service package, such as liver protection drugs, second-line anti-TB drugs, and extra non-recommended lab tests, which were not on the reimbursable list or on the list of free TB services. Therefore, inpatients still needed to pay large amounts of money for the costs out of the service package, and the ratio of OOP costs to total costs was above 40%. Although the inpatients paid large amounts of money out of the service package, the median total cost was 11674.20 CNY, which was still lower than other studies in Jiangsu Province, which was 20411 CNY in Lianyungang City [33] and 15500 CNY in Zhangjiagang City [6].

Results from generalized linear regression analysis showed that the average total costs of smear-positive patients (12384.00 CNY) was higher than that of smear-negative patients (11260.25 CNY). Sputum smear positivity often indicates that the patient’s condition is relatively serious, which has a direct impact on hospitalization costs. In addition, positive sputum smears can affect the length of hospital stay indirectly and increase the total costs of hospitalization. Studies on hospitalization costs for tuberculosis patients have shown that the length of hospital stay has a direct impact on hospitalization costs [34]. Another analysis of hospitalization costs for inpatients with tuberculosis in a designated tuberculosis hospital in Tianjin City in China [35] indicated that the length of hospital stay was the greatest impact on TB patient hospitalization costs. The longer the patient stays in the hospital, the higher the cost.

We observed that occupation was one of the factors associated with total costs and OOP costs. The costs of manual labourers were higher than those of other groups. In our study, most manual labourers, including farmers, rural migrant workers and shepherds, were undereducated. Previous studies suggested that patients with lower education levels were associated with low incomes [36]. Due to an insufficient understanding of tuberculosis and poor incomes, these patients often delay seeking medical care. When they were diagnosed with tuberculosis, the symptoms were often stronger or accompanied by other complications, resulting in high costs of treatment [37]. In a systematic review, Sreeramareddy et al. [38] described the time delay in diagnosis of tuberculosis patients in China as approximately 25 to 714 days. A survey conducted by Dr. Needham [39] in Zambia indicated that patients with a high education level could recognize the symptoms of tuberculosis and seek medical service in time. In addition, most of the manual labourers in our study were covered by URBMI (65.6%), and the reimbursement rate out of the service package was lower than that of other groups, resulting in higher OOP costs than in other groups. The income of manual labourers should also be affected by the loss of work, the costs of diet, transportation, etc. during hospitalization, and the actual economic burden of these patients should be heavier. The OOP cost for students was lower than that of other groups. The reason is that the reimbursement rate of student insurance in many areas of China is higher than that of UEBMI and URBMI. The results of our study also revealed that age was associated with OOP costs. As the patients’ age increased, the ALOS was shortened. The ALOS of the elderly over 60 years was the shortest (23.28 ± 16.19 days), which was 10 days less than that of the other groups, reducing the hospitalization expenses.

Despite some limitations, the key findings of our study can still reveal the issues that tuberculosis patients faced in Zhenjiang City. High out-of-pocket payments can lead to catastrophic health expenditure. Based on the findings, we would like to propose that health policy makers should take further steps to control the high TB patient hospitalization rates and inpatient service expenditure. First, revise the reimbursement policies relevant to TB treatment, expand the service package to include more medical items. Second, formulate TB treatment guideline and supervise its implementation to encourage the designated hospitals to provide necessary medical services and as a result reduce the financial burden on TB patients.

### Study limitations

Some limitations existed in this study. First, we did not obtain all the expenditure records of the 211 inpatients and may not be able to overall reflect the economic burden of inpatients. Second, we only analysed the medical expenditures of inpatients; the expenditures of outpatients, including transportation, accommodation and food, and the loss of income due to the disease were not analysed because of the lack of data collected. High expenditure was associated with companions and transportation [40]. Third, Zhenjiang City has 3 counties, and the data were not obtained in this study. We planned to collect and analyse the data in further study.

## Conclusion

We observed that the admission rate of TB patients in Zhenjiang City was 62.1%. Within the service package, the reimbursement rates achieved 80%, and the medical expenditures for TB were also maintained below 8000 CNY per patient. However, massive expenditures out of the service package still existed. Despite advances in TB insurance policies, there are substantial costs associated with TB diagnosis and treatment, and TB patients still face a heavy financial burden. Health care providers may expand the items included in the service package, and revise the reimbursement policies relevant to TB treatment, to encourage the designated hospitals to provide appropriate medical services and to reduce the financial burden on TB patients.

## Additional files

Additional file 1: Details of the 356 TB patients. These data contain demographic and social characteristics of the 356 TB patients, including gender, age, occupation, patient category, residence, sputum smear test, hospitalization status, type of health insurance, etc. (XLS 70 kb)

Additional file 2: Details of the 221 TB inpatients. These data contain the hospitalization records of 221 inpatients, including hospitalization times, length of hospital stay, and type of health insurance. (XLS 48 kb)

Additional file 3: Medical expenditure of the 141 inpatients. These data contain the social characteristics and hospitalization expenditures of 141 TB inpatients, including total costs and OOP costs. (XLS 44 kb)

## Abbreviations

ALOS: Average length of stay
CDC: Centers for Disease Control and Prevention
CHE: Catastrophic health expenditure
CNY: Chinese Yuan
DOTS: Directly observed treatment short-course
GDP: The gross domestic product
HIS: Hospital Information System
HRS: Hospital Reimbursement System
NCMS: New Rural Cooperative Medical System
NTP: The National Tuberculosis Control Program
OOP: Out-of-pocket
TB: Tuberculosis
TMIS: China TB Management Information System
UEBMI: Urban Employee Basic Medical Insurance scheme
URBMI: Urban Resident Basic Medical Insurance scheme
WHO: World Health Organization
